# “Hot” electrons in metallic nanostructures—non-thermal carriers or heating?

**DOI:** 10.1038/s41377-019-0199-x

**Published:** 2019-10-02

**Authors:** Yonatan Dubi, Yonatan Sivan

**Affiliations:** 1 Department of Chemistry and the Ilse Katz Center for nanoscale Science and Technology, Ben-Gurion University of the Negev, Beer Sheva, Israel; 2 School of Electrical and Computer Engineering and the Ilse Katz Center for nanoscale Science and Technology, Ben-Gurion University of the Negev, Beer Sheva, Israel

**Keywords:** Nanoparticles, Optoelectronic devices and components, Nanophotonics and plasmonics, Electronics, photonics and device physics

## Abstract

Understanding the interplay between illumination and the electron distribution in metallic nanostructures is a crucial step towards developing applications such as plasmonic photocatalysis for green fuels, nanoscale photodetection and more. Elucidating this interplay is challenging, as it requires taking into account all channels of energy flow in the electronic system. Here, we develop such a theory, which is based on a coupled Boltzmann-heat equations and requires only energy conservation and basic thermodynamics, where the electron distribution, and the electron and phonon (lattice) temperatures are determined uniquely. Applying this theory to realistic illuminated nanoparticle systems, we find that the electron and phonon temperatures are similar, thus justifying the (classical) single-temperature models. We show that while the fraction of high-energy “hot” carriers compared to thermalized carriers grows substantially with illumination intensity, it remains extremely small (on the order of 10^−8^). Importantly, most of the absorbed illumination power goes into heating rather than generating hot carriers, thus rendering plasmonic hot carrier generation extremely inefficient. Our formulation allows for the first time a unique quantitative comparison of theory and measurements of steady-state electron distributions in metallic nanostructures.

## Plasmonics: The latest ‘hot’ topic in photo-catalysis

Plasmonic metal nanoparticles are attracting considerable attention as they were claimed to produce high energy non-thermal (so-called ‘hot’) electrons for use in photo-catalytic reactions, such as hydrogen dissociation, water splitting, and artificial photosynthesis. Estimating the number of hot electrons generated by a given level of illumination and disentangling it from mundane heating, however, has remained challenging. Now, Yonatan Dubi and Yonatan Sivan from Ben-Gurion University, Israel, have developed a model for determining the electron distribution in a metal nanostructure under continuous wave illumination, allowing, for the first time, a comparison of heating and non-thermal effects in the steady-state electron distributions in metallic nanostructures. They find that non-thermal carrier generation is an extremely small effect, as essentially all absorbed photon energy gives rise to heating. This finding revolutionizes our understanding of plasmon-assisted photocatalysis experiments.

What happens to electrons in a metal when they are illuminated? This fundamental question has been a driving force in the shaping of modern physics since the discovery of the photoelectric effect. In recent decades, this problem has resurfaced from a new angle, owing to developments in the field of nanoplasmonics^1,2^, where metallic nanostructures give rise to resonantly enhanced local electromagnetic fields, and hence, to controllable optical properties.

Even more recently, there has been growing interest in controlling also the electronic and chemical properties of metal nanostructures. In particular, upon photon absorption, energy is transferred to the electrons in the metal, thus driving the electron distribution out of equilibrium; the generated non-thermal electrons - sometimes (ill)referred to as “hot” electrons - can be exploited for photodetection^3–5^ and up-conversion^6,7^. Many other studies have claimed that “hot” electrons can be exploited in photocatalysis, namely, to drive a chemical reaction such as hydrogen dissociation, water splitting^8–14^ or artificial photosynthesis^15,16^. These processes have immense importance in paving the way towards realistic alternatives to fossil fuels.

Motivated by the large and impressive body of experimental demonstrations of the above-mentioned applications, many theoretical studies address the question: how many non-equilibrium high-energy (“hot”) electrons are generated for a given illumination. Naïvely, one would think that the answer is already well-known, but in fact, finding a quantitative answer to this question is a challenging task. A complete theory of non-equilibrium carrier generation should not only include a detailed account of the non-equilibrium nature of the electron distribution, but also account for the possibility of the electron temperature to increase (via *e* − *e* collisions), the phonon temperature to increase (due to *e* − *ph* collisions), as well as for energy to leak from the lattice to the environment (e.g., a substrate or solution). The model should then be used for finding the steady-state non-equilibrium electron distribution that is established under *continuous wave* (CW) illumination, as appropriate for technologically-important applications such as photodetection and photocatalysis.

Quite surprisingly, to date, there is no comprehensive theoretical approach that takes all these elements into account. Typically, the transient electron dynamics is studied^17–22^, focusing on an accurate description of the material properties, e.g., metal band structure and collision rates^23–25^; some studies also account for the electron temperature dynamics^20,22^ and (to some extent) the permittivity^22^ dynamics. On the other hand, the few pioneering theoretical studies of the steady-state non-equilibrium under CW illumination^26,27^ account for the electron distribution in great detail, but assume that the electron and phonon (lattice) temperatures are *both* at room temperature. In^28^ an “effective” electron temperature is referred to (without formally defining it); it is assumed to be higher than the environment temperature, but is pre-determined (rather than evaluated self-consistently). As discussed in SI Section S3, the chosen values for that “effective” electron temperature are questionable and while it is claimed in^29^ that *T*_*ph*_ > *T*_*env*_, there is no indication of this in the published papers.

The fact that the phonon and electron temperatures were not calculated in previous theoretical studies of the “hot electron” distribution is not a coincidence. After all, the system is out of equilibrium, so how can one define a unique value for the temperature, which is inherently an equilibrium property^30^? Yet, it is well-known that the temperature of metallic nanostructures *does* increase upon CW illumination, sometimes to the degree of melting (or killing cancer cells); this process is traditionally described using classical, single-temperature heat equations (see, e.g.,^31–33^).

Here, we suggest a unique self-contained theory for the photogeneration of non-equilibrium energetic carriers in metal nanostructures that reconciles this “paradox”. The framework we chose is the quantum-like version of the Boltzmann equation (BE), which is regularly used for describing electron dynamics in metallic systems more than a few nm in size^17–20,22,34–39^. We employ the relaxation time approximation for the electron-electron thermalization channel to determine the electron temperature without ambiguity. Furthermore, on top of the BE we add an equation for the phonon temperature such that together with the integral version of the BE, our model equations provide a microscopic derivation of the extended two-temperature model^32,33^. In particular, the electron and phonon temperatures are allowed to rise above the ambient temperature and energy can leak to the environment while energy is conserved in the photon-electron-phonon-environment system. The latter aspect distinguishes our calculation of the steady-state non-equilibrium from previous ones.

Using our theory, we show that the population of non-equilibrium energetic electrons and holes (i.e., negative values of the deviation from thermal equilibrium (*f* − *f*^*T*^, see below) see e.g.,^36^). can increase dramatically under illumination, yet this process is *extremely inefficient*, as almost all the absorbed energy leads to heating; the electron and phonon temperatures are found to be essentially similar, thus, justifying the use of the classical single-temperate heat model^31^. Somewhat surprisingly, we find that just above (below) the Fermi energy, the non-equilibrium consists of holes (electrons) rather than the other way around; we show that this behaviour is due to the dominance of *e* − *ph* collisions in that electron energy regime. All these results are very different from those known for electron dynamics under ultrafast illumination, as well as from previous studies of the steady-state scenario that did not account for all three energy channels (e.g.,^26–28,40^). A detailed comparison to earlier work is presented throughout the main text and the supplementary information (SI).

## Model

We start by writing down the Boltzmann equation in its generic form,

1 $$\frac{\partial f\left({\cal{E}},T_e,T_{ph} \right)}{\partial t} = \left(\frac{\partial f}{\partial t} \right)_{\!ex} + \left(\frac{\partial f}{\partial t} \right)_{\!e - e} + \left( \frac{\partial f}{\partial t} \right)_{\!e - ph}$$

Here, *f* is the electron distribution function at an energy ${\mathcal{E}}$, electron temperature *T*_*e*_ and phonon temperature *T*_*ph*_, representing the population probability of electrons in a system characterized by a continuum of states within the conduction band; finding it for electrons under CW illumination is our central objective. For simplicitiy, and with essentially no loss of accuracy, we neglect the deviation of the phonon system from thermal equilibrium. This is an assumption that was adopted in almost all previous studies on the topic; accounting for the phonon non-equilibrium can be done in a way similar to our treatment of the electron non-equilibrium, see e.g., ref. ^20,41^.

**Fig. 1:**
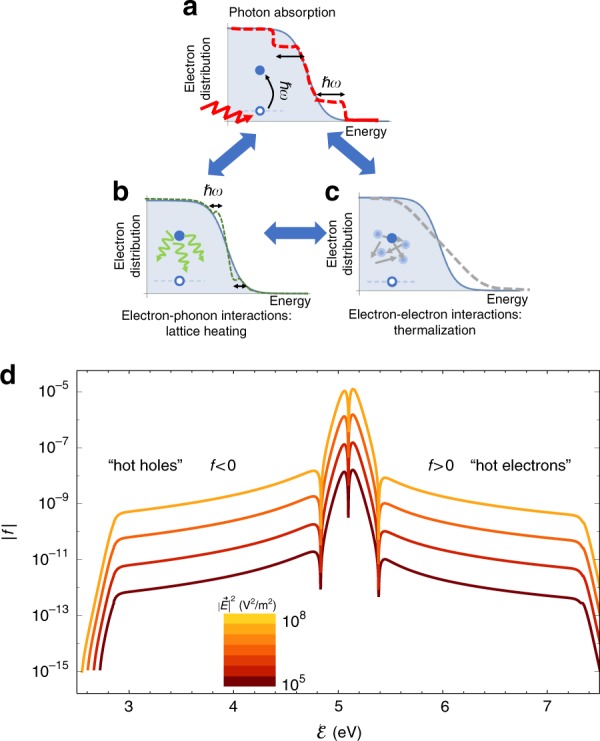
Full non-equilibrium electron distribution under illumination. The steady-state of the system is determined by the balance of three processes, shown in the background of the thermal distribution (grey). **a** Absorption of photons by an electron, with an energy quanta *ħω*. **b** Electron - phonon scattering, which leads to lattice heating. **c** Electron-electron scattering, which leads to thermalization and electron heating. In addition, the excess thermal energy from the lattice can be transferred to the environment. **d** Deviation from the equilibrium distribution at the ambient electron temperature, namely, ${\mathrm{\Delta }}f \equiv f({\cal{E}},T_e,T_{ph}) - f^T({\cal{E}},T_{env})$, as a function of electron energy for various incoming field levels; the system is a bulk Ag illuminated by *ħω* = 2.25 eV photons, see all parameter values in Table S1. Non-thermal hole densities, which correspond to Δ*f* < 0, are shown for simplicity in opposite sign. The dashed vertical line represents the Fermi energy. The various dips are artefacts of the semilogarithmic scale—they represent sign changes of Δ*f*

The right-hand side of the BE describes three central processes, which determine the electron distribution. Electron excitation due to photon absorption increases the electron energy by *ħω*, thus, generating an electron and a hole, see Fig. 1a and Fig. S1a; it is described (via the term $\left( {{\textstyle{{\partial f} \over {\partial t}}}} \right)_{ex}$) using an improved version of the Fermi golden rule type form suggested in^18,19,22,39^ which explicitly incorporates the absorption lineshape of the nanostructure, see Eq. (S9) the discussion in SI Section S1A.

Electron-phonon (*e* − *ph*) collisions cause energy transfer between the electrons and lattice; they occur within a (narrow) energy window (the width of which is comparable to the Debye energy) near the Fermi energy, see Fig. 1b and Fig. S1b. They are described using a general Bloch-Boltzmann-Peierls form^18,34,42^, see the discussion in SI Section S1B.

Electron-electron (*e* − *e*) collisions lead to thermalization. They occur throughout the conduction band but are strongly dependent on the energy—for carrier energies close to the Fermi energy, they are relatively slower than for electrons with energies much higher than the Fermi energy, which can be as fast as a few tens of femtoseconds; see Fig. 1c and^20,22,43^. Traditionally, two generic models are used to describe *e* − *e* collisions. The exact approach involves the 4-body interactions between the incoming and outgoing particles within the Fermi golden rule formulation; see e.g.,^17–20,34,37,42^. This approach has two main drawbacks—first, evaluation of the resulting collision integrals is highly time-consuming^19^; second, it is not clear what is the state into which the system wishes to relax (although it is clear that it should flow into a Fermi distribution at equilibrium conditions).

A popular alternative is to adopt the so-called relaxation time approximation, whereby it is assumed that the non-equilibrium electron distribution relaxes to a Fermi-Dirac form $f^T({\cal{E}},T_e)$^17,28,34,37^ with a well-defined temperature *T*_*e*_, namely, $\left( {\frac{{\partial f({\cal{E}})}}{{\partial t}}} \right)_{e - e} = - \frac{{f - f^T(T_e)}}{{\tau _{e - e}({\cal{E}})}}$, where $\tau _{e - e}({\cal{E}})$ is the electron collision time. The electron temperature that characterizes that Fermi-Dirac distribution is the temperature that the electron subsystem will reach if the illumination is stopped and no additional energy is exchanged with the phonon subsystem. The relaxation time approximation is known to be an excellent approximation for small deviations from equilibrium (especially assuming the collisions are elastic and isotropic^44^). In this approach, the *e* − *e* collision integral is simple to compute, and the physical principle that is hidden in the full collision integral description, namely, the desire of the electron system to reach a Fermi-Dirac distribution, is illustrated explicitly. Most importantly, the relaxation time approximation allows us to eliminate the ambiguity in the determination of the temperature of the electron subsystem. The collision time itself $\tau _{e - e}({\cal{E}})$ is evaluated by fitting the standard expression from Fermi-liquid theory to the computational data of ref. ^22^, see SI Section S1C.

What remains to be done is to determine *T*_*ph*_; it controls the rate of energy transfer from the electron subsystem to the phonon subsystem, and then to the environment. Recent studies of the steady-state non-equilibrium in metals (e.g.,^26–28^) relied on a fixed value for *T*_*ph*_ (choosing it to be either identical to the electron temperature, or to the environment temperature, see discussion above) and/or treated the rate of *e* − *ph* energy transfer using the relaxation time approximation with an *e* − *ph* collision rate that is independent of the field and particle shape. While these approaches ensure that energy is conserved in the electron subsystem, they ignore the dependence of the energy transfer to the environment on the nanoparticle shape, the thermal properties of the host material, the electric field strength and the temperature difference between electrons and phonons. Therefore, these phenomenological approaches fail not only to ensure energy conservation in the complete system (photons, electrons, phonons and environment), but they also fail to provide a correct quantitative prediction of the electron distribution near the Fermi energy (which is strongly dependent on *T*_*ph*_) and provides incorrect predictions regarding the role of the nanoparticle shape and of the host properties on the steady-state electron distribution and the temperatures (see further discussion in ref. ^45^).

To determine *T*_*ph*_ self-consistently while ensuring energy conservation, one must account for the “macroscopic” properties of the problem. Specifically, we multiply Eq. (1) by the product of the electron energy ${\cal{E}}$ and the density of electron states $\rho _e({\cal{E}})$ and integrate over the electron energy. The resulting equation describes the dynamics of the energy of the electrons,

2 $$\frac{{d{\cal{U}}_e}}{{dt}} = W_{ex} - W_{e - ph}$$

Equation (2) has a simple and intuitive interpretation: the dynamics of the electron energy is determined by the balance between the energy that flows in due to photoexcitation ($W_{ex} \equiv {\int} {\cal{E}} \rho _e({\cal{E}})\left( {{\textstyle{{\partial f} \over {\partial t}}}} \right)_{ex}d{\cal{E}}$) and the energy that flows out to the lattice ($W_{e - ph} \equiv - {\int} {\cal{E}} \rho _e({\cal{E}})\left( {{\textstyle{{\partial f} \over {\partial t}}}} \right)_{e - ph}d{\cal{E}}$; see SI Section S1 B).

Similar to Eq. (2), the total energy of the lattice, ${\cal{U}}_{ph}$, is balanced by the heat flowing in from the electronic system and flowing out to the environment, namely,

3 $$\frac{{d{\cal{U}}_{ph}}}{{dt}} = W_{e - ph} - G_{ph - env}(T_{ph} - T_{env})$$

Here, *T*_*env*_ is the temperature of the environment far from the nanostructure and *G*_*ph*−*env*_ is proportional to the thermal conductivity of the environment; it is strongly dependent on the nanostructure geometry (e.g., exhibiting inverse proportionality to the particle surface area for spheres).

Equations (1)–(3) provide a general formulation for the non-thermal electron generation, electron temperature and lattice temperature in metal nanostructures under arbitrary illumination conditions; see also the discussion in SI Section S3. Once a steady-state solution for these equations is found, energy conservation is ensured—the power flowing into the metal due to photon absorption is exactly balanced by heat leakage to the environment. Within the relaxation time approach, there is only one pair of values for the electron and phonon temperatures for which this occurs. Our “macroscopic” approach thus allowed us to determine the temperatures in a system that is out of equilibrium in a unique and unambiguous way. The equations require as input the local electric field distribution from a solution of Maxwell’s equations for the nanostructure of choice; see SI Section S3. In what follows, we numerically search for the steady-state (*∂*/*∂t* = 0) solution of these (nonlinear) equations for the generic (and application-relevant) case of CW illumination. For concreteness, we chose parameters for Ag, taken from a comparison to experiments of ultrafast illumination^18^; the photon energy and local field values are chosen to coincide with the localized plasmon resonance of a Ag nano-sphere in a high permittivity dielectric, similar to many experiments^15,46^ (see Table S1); in particular, the local field in this configuration gives a plasmonic near-field enhancement of at least an order of magnitude, depending on the geometry and material quality. Our approach can be applied to any other configuration just by scaling the local field appropriately; see SI Section S3. This configuration also justifies the neglect of interband transitions (see the discussion in SI Section S1) and field inhomogeneities (see the discussion in SI Section S3). As we demonstrate, this generic case leads to several surprising qualitative new insights, as well as to quantitative predictions of non-equilibrium carrier distributions.

## Results

### Electron distribution

Figure 1d shows the deviation of the electron distribution from the distribution at the ambient temperature (i.e., in the dark), ${\mathrm{\Delta }}f \equiv f({\cal{E}},T_e,T_{ph}) - f^T({\cal{E}},T_{env})$, as a function of electron energy for various local field levels. The distributions depend on the local field quantitatively, but are qualitatively similar, showing that the resonant plasmonic near-field enhancement can indeed be used to increase the number of photogenerated “hot” electrons, as predicted and observed experimentally.

**Fig. 2:**
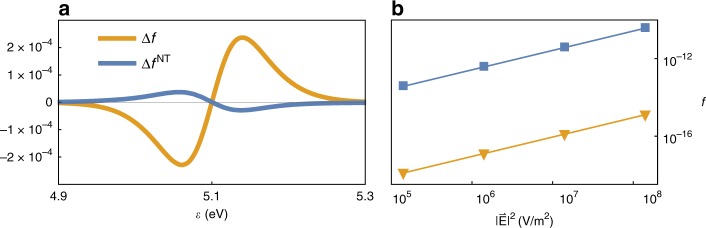
Non-thermal contribution to non-equilibrium. **a** Comparison of the true non-equilibrium distribution ${\mathrm{\Delta }}f^{NT} \equiv f({\cal{E}},T_e,T_{ph}) - f^T({\cal{E}},T_e)$ with Δ*f* within the energy range close to the Fermi energy for $|\vec E|^2 = 10^9[V/m]$. The true non-equilibrium is smaller and of opposite sign, indicating the presence of non-thermal holes (electrons) above (below) the Fermi energy. **b** The populations $f({\cal{E}})$ of electrons at ${\cal{E}} = 1.8$ eV above the Fermi level (blue rectangles) and electrons at ${\cal{E}} = 2.5$ eV (>*ħω*) above the Fermi level (yellow triangles), all as a function of local field, showing a quadratic dependence between the illumination field and “hot” carrier population (with a similar slope)

The overall deviation from equilibrium (see scale in Fig. 1d) is minute, thus, justifying a-posteriori the use of the relaxation time approximation; in fact, near the Fermi energy, the deviation takes the regular thermal form, namely, it is identical to the population difference between two thermal distributions, thus justifying the assignment of the system with electron and phonon temperatures. In particular, the change in population is largest near the Fermi energy; specifically, Δ*f* > 0 (<0) above (below) the Fermi energy, corresponding to electrons and holes, respectively (note that since Δ*f* (and Δ*f*^*NT*^ below) is not a distribution, but rather, a difference of distributions, it can attain negative numbers, representing holes), see Fig. 2a. This is in accord with the approximate (semi-classical) solution of the Boltzmann equation (see e.g., ref. ^34,37^) and the standard interpretation of the non-equilibrium distribution (see e.g., ref. ^40,47^).

### The “true” non-thermal distribution

It is clear that the distributions Δ*f* in Fig. 1 mix the two components of the electron distribution, namely, the thermal and non-thermal parts. To isolate the non-thermal contribution, one should consider the deviation of the electron distribution from the distribution at the *steady-state* temperature, ${\mathrm{\Delta }}f^{NT} \equiv f({\cal{E}},T_e,T_{ph}) - f^T({\cal{E}},T_e)$. Simply put, this is the “true” non-thermal part of the steady-state electron distribution, loosely referred in the literature as the “hot electron distribution”.

Since the differences between $f^T({\cal{E}},T_e)$ and $f^T({\cal{E}},T_{env})$ occur mostly around the Fermi energy, it is instructive to study Δ*f*^*NT*^ in two energy regimes. First, Fig. 2a shows that near the Fermi energy, the population change is now approximately an order of magnitude smaller and of the opposite sign (in comparison to Δ*f*, Fig. 1d). This is a somewhat surprising result, which means that the non-thermal distribution just above (below) the Fermi energy is characterized by the presence of non-thermal holes (electrons). This result could be obtained only when the explicit separation of the three energy channels is considered, allowing *T*_*e*_ to increase above *T*_*env*_. Notably, this is the exact opposite of the regular interpretation of the non-equilibrium distribution (as e.g., in Fig. 1d and standard textbooks^34,37^), which results from a calculation that *does not* account for the electron temperature increase. From the physical point of view, this change in sign originates from *e*−*ph* collisions, as it has the same energy-dependence as the Bloch-Boltzmann-Peierls term; compare Fig. 2a with Fig. S1b.

Second, further away from the Fermi energy, *ħω*-wide (roughly symmetric) shoulders are observed on both sides of the Fermi energy (Fig. 1d), corresponding to the generation of non-thermal holes (Δ*f*^*NT*^ < 0) and non-thermal electrons (Δ*f*^*NT*^ > 0). These high-energy charge carriers are those referred to in the context of catalysis of chemical reactions.

For energies beyond *ħω* from the Fermi energy, the non-thermal distribution is much lower, as it requires multiple photon absorption (observing the expected multiple step structure^20^ is numerically very challenging for the steady-state case). This implies that in order to efficiently harvest the excess energy of the non-thermal electrons, one has to limit the harvested energy to processes that require an energy smaller than *ħω*.

The non-thermal electron distributions we obtained look similar to those obtained by calculations of the excitation rates due to photon absorption^23,24,40,47^. However, as pointed out in^23,28^, that approach yields the correct electron distribution only immediately after illumination by an ultrashort pulse (essentially before any electron scattering processes take place); this distribution would be qualitatively similar to the steady-state distribution only if all other terms in the BE were energy-independent; as explained in SI Section S1 and seen from Fig. S1, this is not the case. More specifically, this approach does not correctly predict the electron distribution near the Fermi energy; this means that the total energy stored in the electron system is not correctly accounted for and that the contribution of interband transitions to the non-equilibrium cannot be correctly determined. The main reason for these inaccuracies is that these studies did not correctly account for the electron and phonon temperatures, hence, for the energy flow from the thermal electrons to the lattice. As a result, quantitative conclusions on the distribution drawn in these studies are incorrect. Similar inaccuracies are also found in the calculations of^21,26–28^.

On the other hand, these approaches can be used to provide a quantitative prediction of the electron distribution away from the Fermi energy, where *e*−*ph* interactions are negligible (see ref. ^45^, Section IIB); peculiarly, however, this was not attempted previously^23,24,40,47^, and instead, only claims about the qualitative features of the electron distributions were made. In this vein, our calculations also show that the number of photogenerated high-energy electrons Δ*f*^*NT*^ is independent of *G*_*ph*−*env*_ (see Fig. S5 and the discussion in SI Section S3). Since *G*_*ph*−*env*_ is proportional to the thermal conductivity of the host and inversely proportional to the particle surface area, this implies that if a specific application relies on the number of high-energy electrons, then, it will be relatively insensitive to the thermal properties of the host and the particle size. Conversely, since the temperature increase is inversely proportional to *G*_*ph*−*env*_ (see ref. ^48^ and Fig. S5), the difference in the photocatalytic rate between the TiO_2_ and SiO_2_ substrates (compare ref. ^46^ and ref. ^49^) is likely a result of a mere temperature increase, but is unlikely to be related to the number of photogenerated high-energy electrons (see further discussion in ref. ^50^).

### Electron and phonon temperatures

**Fig. 3:**
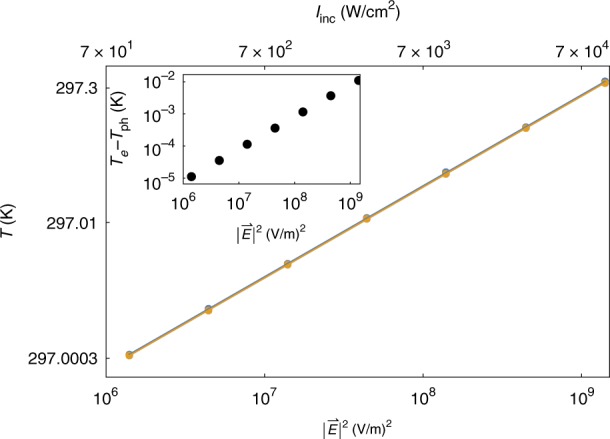
Temperature increase under illumination. The electron (blue) and lattice (orange) temperatures extracted from the data of Figs. 1 and 2 as a function of the local field (loglog scale), showing a linear dependence on the field-squared (top *x*-axis shows the corresponding incident intensity). Inset: difference between electron and phonon temperatures as a function of the field-squared, showing that (i) the difference is also linear, and (ii) several orders of magnitude smaller than the temperatures themselves (making the electron and phonon temperatures essentially equal)

As noted above, our approach also allows a quantitative estimate of both electron and phonon temperatures. In Fig. 3, these are plotted (on a log-log scale) as a function of the local field-squared $|\vec E|^2$ (also translated into incident illumination intensity *I*_*inc*_ in the upper x-axis for the specific case of a 5nm Ag sphere). As observed, both temperatures grow linearly with $|\vec E|^2$ over many decades of the field, as in the classical (single-temperature) approach^31,33^. This is a nontrivial result, since our non-equilibrium model equations exhibit an implicit nonlinear dependence on the temperatures. Figure 3 also shows that *T*_*e*_ is only slightly higher than *T*_*ph*_. This provides the first (qualitative and quantitative) justification, to the best of our knowledge, for the use of the single-temperature heat equation in the context of metallic nanostructures under illumination^31,33^; more generally, it provides a detailed understanding of the origin of the single-temperature model, as well as the limits to its validity (as at high intensities the electron-phonon temperature difference may become substantial).

### Efficiency

**Fig. 4:**
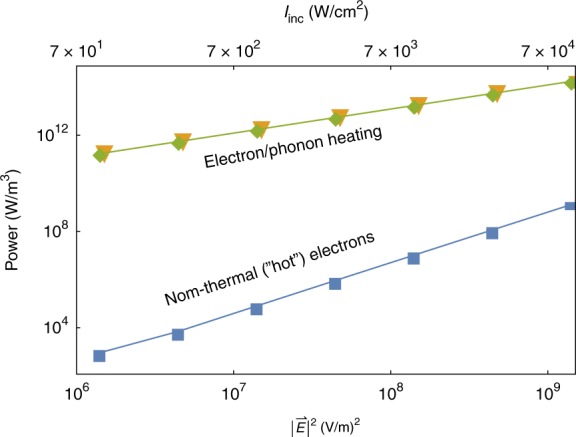
Power density and its distribution between the different channels. Power densities going into the thermal electron and lattice systems (*W*_*e*−*e*_ in green diamonds and *W*_*e*−*ph*_ in orange triangles, respectively), compared with the power going to the non-thermal electrons ($W_{ex}^{NT}$ in blue squares), all as a function of the local field. The power fraction that flows into the thermal channels (i.e. to heat the systems) is substantially larger than that going into generating non-thermal electrons

Our approach allows us to deduce how the power density pumped into the metal by the absorbed photons splits into the non-thermal electrons and into heating the electrons and the phonons (see Fig. 4), providing a way to evaluate the efficiency of the non-thermal electron generation (detailed calculation described in SI Section S1 D). Remarkably, one can see that the overall efficiency of the non-thermal electron generation is truly abysmal: At low intensities, the power channelled to the deviation from equilibrium ($W_{ex}^{NT} \equiv {\int} {\cal{E}} \rho _e({\cal{E}})\left( {{\textstyle{{\partial f^{NT}} \over {\partial t}}}} \right)_{ex}d{\cal{E}}$) is more than 8(!) orders of magnitude lower than the power invested in the heating of the electrons and phonons (which are accordingly nearly similar). This is in correlation with the results of Fig. 1: most absorbed power leads to a change in the electron distribution near the Fermi energy rather than to the generation of high-energy electrons, as one might desire. This shows that any interpretation of experimental results that ignores electron and phonon heating should be taken with a grain of salt. It is thus the main result of the current study. This shows, in particular, that even the pessimistic estimates in ref. ^51^ on the numbers of high-energy non-thermal electrons were too optimistic.

The performance of a “hot” electron system (e.g., for catalysis or photodetection, when electrons need to tunnel out of the nanoparticle) is essentially proportional to the electron distribution at the relevant energies (see SI Section S2). A comparison with the pure thermal distribution of high-energy electrons (Fig. 2) shows that the absolute electron population can be many orders of magnitude higher compared to the thermal distribution at the steady-state temperature. Such an enhancement was indeed observed in “hot” electron based photodetection devices^5,52^, but not in “hot” electron photocatalysis^8,9,11–13,21,40,53^. This result underlies the alternative purely-thermal interpretation of plasmon-assisted photocatalysis experiments we proposed in^45,50,54^.

One can identify several pathways towards significant improvements in the efficiency of photogeneration of non-thermal electrons. In particular, as seen in Fig. 4, as the local field is increased, the power fraction going to non-equilibrium increases to 10^−5^. This improvement motivates the study of the non-thermal electron distribution for higher intensities. Such study, however, will require extending the existing formulation by extracting self-consistently also the metal permittivity from the non-equilibrium electron distribution *f* (as done above for the electron temperature). Other pathways for improved “hot” electron harvesting may rely on interband transitions due to photons with energies far above the interband threshold^24,55^, or optimizing the nanostructure geometry to minimize heating and maximize the local fields^56^, e.g., using few-nm particles (which support the same number of non-thermal carriers but lower heating levels).

Finally, the formulation we developed serves as an essential first step towards realistic calculations of the complete energy harvesting process, including especially the tunnelling process, and the interaction with the environment, be it a solution, gas phase or a semiconductor. Our formulation enables a quantitative comparison with experimental studies of all the above processes and the related devices. Similarly, our formulation can be used to separate thermal and non-thermal effects in many other solid-state systems away from equilibrium, particularly, semiconductor-based photovoltaic and thermo-photovoltaic systems.

## Supplementary information

SUPPLEMENTARY INFORMATION for “Hot” electrons in metallic nanostructures - non-thermal carriers or heating?
