# Supplementary material for: “Hot” electrons in metallic nanostructures—non-thermal carriers or heating?
Source: Light Sci Appl. 2019 Oct 2;8:89. doi: 10.1038/s41377-019-0199-x (PMC6804576; doi:10.1038/s41377-019-0199-x)
Supplement: Supplementary file 1 — SUPPLEMENTARY INFORMATION for “Hot” electrons in metallic nanostructures - non-thermal carriers or heating? [file 41377_2019_199_MOESM1_ESM.pdf]

Supplementary Information:
“Hot” electrons in metallic nanostructures - non-thermal carriers or heating?

Yonatan Dubi

*Department of Chemistry and the Ilse Katz Center for nanoscale Science and Technology, Ben-Gurion University of the Negev, Beer Sheva, Israel**

Yonatan Sivan

School of Electrical and Computer Engineering, Ben-Gurion University of the Negev and the Ilse Katz Center for nanoscale Science and Technology, Ben-Gurion University of the Negev, Beer Sheva, Israel†

(Dated: August 29, 2019)

*Electronic address: jdubi@bgu.ac.il

†Electronic address: sivanyon@bgu.ac.il

I. SOLUTION OF THE QUANTUM-LIKE BOLTZMANN EQUATION

We determine the electron distribution in the conduction (sp) band, $f(\mathcal{E}, T_e, T_{ph})$, in a metal nanostructure under continuous wave (CW) illumination by solving the quantum-like Boltzmann equation (BE). This model is in wide use for such systems [1–8]; It is valid for nanoparticles which are more than a few nm in size (hence, not requiring energy discretization) [9, 10] and for systems where coherence and correlations between electrons are negligible. The latter assumption holds for a simple metal at room temperatures (or higher), as it has a large density of electrons and fast collision mechanisms. In order to include quantum finite size effects or quantum coherence effects, one can use the known relation between the discretized BE and quantum master equations [11, 12] or by replacing the BE by that equation [10, 13, 14].

For simplicity, we consider a quasi-free electron gas such that the conduction band is purely parabolic (with a Fermi energy of $\mathcal{E}_F = 5.1$eV and total size of $\mathcal{E}_{max} = 9$eV, typical to Ag, see [2]). This allows us to represent the electron states in terms of energy $\mathcal{E}$ rather than momentum. We also neglect interband (*d* to *sp*) transitions - these have a small role when describing metals like Al illuminated by visible light, Ag for wavelengths longer than about 500nm or so, or Au for near infrared frequencies, where a dominantly Drude response is exhibited. Furthermore, as noted in [10], interband transitions are not likely to generate electrons with energies far above the Fermi level unless the photon energy is much higher than the bandgap energy.

The resulting Boltzmann equation is

$$\frac{\partial f\left(\mathcal{E}(\vec{k}); T_e, T_{ph}\right)}{\partial t} = \underbrace{\left(\frac{\partial f}{\partial t}\right)_{ex}}_{photon\ absorption} + \underbrace{\left(\frac{\partial f}{\partial t}\right)_{e-ph}}_{e-ph\ collisions} + \underbrace{\left(\frac{\partial f}{\partial t}\right)_{e-e}}_{e-e\ collisions}, \tag{S1}$$

where f is the electron distribution function at an energy $\mathcal{E}$, electron temperature T_e and phonon temperature T_{ph}, representing the population probability of electrons in a system characterized by a continuum of states within the conduction band. The first term on the right-hand-side (RHS) of Eq. (S1) describes excitation of conduction electrons due to photon absorption, see SI Section I A below for its explicit form. The second term on the RHS of Eq. (S1) describes energy relaxation due to collisions between electrons and phonons, see SI Section I B below for its explicit form. This interaction makes the electrons in our model

only quasi-free. The third term on the RHS of Eq. (S1) (see SI Section I C below for its explicit form) represents the thermalization induced by $e-e$ collisions, i.e., the convergence of the *non*-thermal population into the *thermalized* Fermi-Dirac distribution, given by

$$f^T(\mathcal{E};T_e) = \left(1 + e^{(\mathcal{E}-\mathcal{E}_F)/k_B T_e}\right)^{-1}, \tag{S2}$$

where k_B is the Boltzmann constant [15].

Note that our model does not require indicating what is the exact nature of the various collisions (Landau damping, surface/phonon-assisted, etc., see discussions in [9, 16–18]), but rather, it accounts only for their cumulative rate. Within this description, it was shown in [9, 16] that the total electron collision time is independent of the size of the metal nanoparticle [19]. Our model also does not account for electron acceleration due to the force exerted on them by the electric field (which involves a classical description, see SI Section I A below), nor for drift due to its gradients or due to temperature gradients; these effects will be small in the regime of intensities considered in our study, especially for few nm (spherical) particles (see also SI Section III below) [20, 21]. Similar simplifications were adopted in most previous studies of this problem, e.g., [9, 10, 13, 14, 22, 23]). These neglected effects can be implemented in our formalism in a straightforward way.

Finally, we emphasize that the results shown in the main text are not sensitive to the details of the general model. In fact, our procedure can be made more system specific; for instance, the metal band structure can be taken into account [16, 18], few nm nanoparticles can be studied by writing the BE in momentum space and discretizing it [9], and further quantum effects may be considered by replacing the BE by a quantum master equation [10, 13, 14]. We do not expect any such change to have more than a moderate quantitative effect on the results shown below.

The steady-state solution of Eqs. (1)-(3) was attained numerically by writing the (thermal) electron and phonon energies as the product of the corresponding heat capacities and temperatures (see SI Section I D) and letting the system evolve naturally to the steady-state by ramping up slowly the electric field. Table I shows the values of all parameters used in our simulations. We observe that the results are insensitive to the initial conditions and choice of various parameter values.

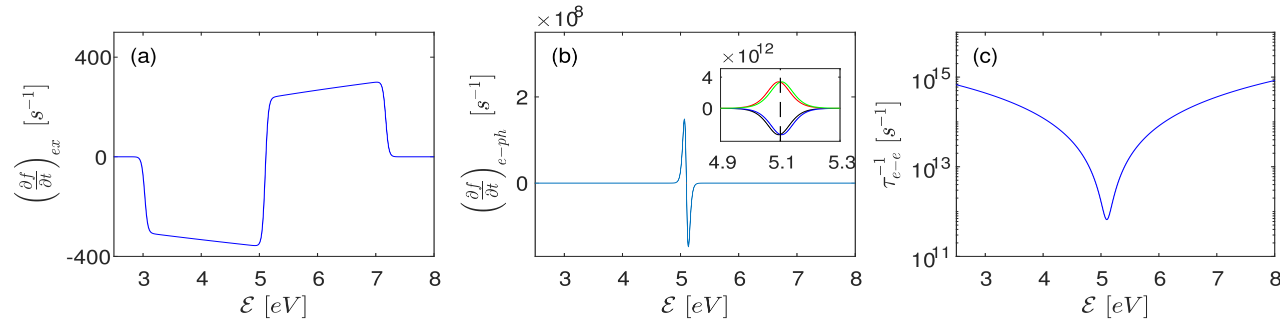

FIG. S1: (Color online) (a) $\left(\frac{\partial f}{\partial t}\right)_{ex}$ (S9) as a function of electron energy for a local field of $|\vec{E}| = 7 \cdot 10^3 V/m$. (b) The $e-ph$ collision rate (S10) as a function of electron energy for $T_e - T_{ph} = 0.2$°K. The inset shows the four competing phonon generation/absorption processes. (c) The $e-e$ collision rate as a function of electron energy, as given by Fermi liquid theory, Eq. (S11).

A. The quantum mechanical excitation term

Usually, the BE is regarded as a (semi-)classical model of electron dynamics. Indeed, several popular textbooks draw the links between the BE to the classical model of an electron motion in an electric field (e.g., [1, 2, 25, 26]). In this case, the change of momentum of the electrons (acceleration) due to the force exerted on them by the electric field corresponds to a *coherent* excitation term, i.e., a term which is proportional to $\frac{\partial}{\partial \mathcal{E}} \frac{\partial \mathcal{E}}{\partial \vec{k}} \cdot \frac{\partial \vec{k}}{\partial t} \sim \vec{v} \cdot \vec{E}$. However, since it relies on a classical field, this expression describes the photon-electron interaction correctly only if the energy imparted on the electron by the electric field is much greater than the energy of a single photon [27]. Since this is not the case, this term does not allow one to derive correctly the non-equilibrium distribution; in fact, this failure to produce experimental observations triggered Einstein to employ a quantized model for the photo-electric effect, and eventually led to the creation of quantum mechanics theory, as we know it.

TABLE S1: Parameters used in the simulations; values chosen for (low quality [24]) 5nm Ag sphere.

parameter	parameter symbol	value
photon wavelength	λ	2.25eV
metal permittivity	$\epsilon_{Ag}(\lambda)$	$-8.5 + 1.8i$ [24]
host permittivity	ϵ_h	4.25
Fermi energy	$\mathcal{E}_F$	5.1eV
conduction band width	$\mathcal{E}_{max}$	9eV
chemical potential	μ	5.1eV
ph-env coupling	G_{ph-env}	$5 \cdot 10^{14} W/m^3K$
electron density	n_e	$5.86 \cdot 10^{28} m^{-3}$
speed of sound	v_{ph}	3650 m/s
environment temperature	T_{env}	$297K$
electron mass	m_e	$9.1 \cdot 10^{31}$ kg

In order to circumvent this problem *within* the BE, frequently the (semi-)classical (linear ($\sim \vec{E}$), coherent) excitation term is replaced by a quantum-like ($\sim |\vec{E}|^2$, incoherent) term derived from the Fermi golden rule [5–9, 23]. Early derivations of this term (e.g. [5]) did not supply a rigorous expression for its magnitude, but rather fit its magnitude to experimental results. Later studies attempted to link the magnitude of this term to the total absorbed power [7]. A systematic derivation was provided in [9].

Here, we employ the simpler, elegant expression proposed in [8], namely, we define $A(\mathcal{E};\omega)$ such that $A(\mathcal{E};\omega)d\omega d\mathcal{E}$ is the (joint) probability of photon absorption of frequency between ω and $\omega + d\omega$ for final energy $\mathcal{E}$ measured with respect to the bottom of the band at $\mathcal{E} = 0$. We define this probability as

$$A(\mathcal{E}_{final} = \mathcal{E};\omega) = \frac{n_A(\omega)}{N_A} \frac{D_J(\mathcal{E},\mathcal{E}-\hbar\omega)\rho_J(\mathcal{E},\mathcal{E}-\hbar\omega)}{\int D_J(\mathcal{E},\mathcal{E}-\hbar\omega)\rho_J(\mathcal{E},\mathcal{E}-\hbar\omega)d\mathcal{E}}, \tag{S3}$$

where $D_J(\mathcal{E}_{final},\mathcal{E}_{initial})$ is the squared magnitude of a transition matrix element for the electronic process $\mathcal{E}_{initial} \to \mathcal{E}_{final}$; Further, ρ_J is the population-weighted density of pair states,

$$\rho_J(\mathcal{E}_{final},\mathcal{E}_{initial}) = \left[f(\mathcal{E}_{initial})\rho_e(\mathcal{E}_{initial})\right]\left[(1 - f(\mathcal{E}_{final})\rho_e(\mathcal{E}_{final})\right], \tag{S4}$$

and $\rho_e = \frac{3n_e}{2\mathcal{E}_F}\sqrt{\frac{\mathcal{E}}{\mathcal{E}_F}}$ is the density of states of a free electron gas [2], n_e being the electron

density. Finally, $n_A(\omega)$ is the number density of absorbed $\hbar\omega$ photons per unit time between ω and $\omega + d\omega$ and $N_A = \int d\omega n_A(\omega)$ is the total number density of absorbed photons per unit time. For CW illumination, it is given by

$$N_A = \frac{\langle p_{abs}\left(\vec{r}, t\right)\rangle_t}{\hbar\omega}, \tag{S5}$$

where the absorbed optical power density (in units of W/m^3) is given by the Poynting vector [28], namely,

$$\langle p_{abs}\left(t\right)\rangle_t = \omega\epsilon''(\omega, T_e, T_{ph})\langle\vec{E}(t)\cdot\vec{E}(t)\rangle_t, \tag{S6}$$

where the temporal averaging, $\langle\rangle_t$, is performed over a single optical cycle such that only the time-independent component remains. Note that the absorption lineshape arises naturally from the spectral dependence of the local electric field in Eq. (S6); it depends on the nanostructure geometry and the permittivities of its constituents. This way, there is no need to introduce the lineshape phenomenologically as done in [8].

The absorption probability of a $\hbar\omega$ photon, A (S3), satisfies

$$\int_0^\infty A(\mathcal{E};\omega)d\mathcal{E} = \frac{n_A(\omega)}{N_A}, \tag{S7}$$

and the net change of electronic population at energy $\mathcal{E}$ per unit time and energy at time t due to absorption is $N_A\phi_A$, where

$$\phi_A(\mathcal{E};\omega) = \int_0^\infty d\omega[A(\mathcal{E};\omega) - A(\mathcal{E}+\hbar\omega;\omega)], \tag{S8}$$

is a quantity describing the total (probability of a) population change at energy $\mathcal{E}$ per unit time and energy at time t.

Altogether, the change of population due to photon excitation is given by

$$\left(\frac{\partial f}{\partial t}\right)_{ex}(\mathcal{E}) = \frac{N_A\phi_A(\mathcal{E})}{\rho_e(\mathcal{E})}, \tag{S9}$$

so that electron number conservation is ensured, $\int d\mathcal{E}\rho_e(\mathcal{E})\left(\frac{\partial f}{\partial t}\right)_{ex}(\mathcal{E}) \sim \int d\mathcal{E}\phi_A(\mathcal{E}) = 0$.

The functional form of Eq. (S9) is shown in Fig. S1(a) - one can see a roughly flat, $\hbar\omega$-wide region of positive rate above the Fermi energy, and a corresponding negative regime below the Fermi energy. In that regard, the incoherent, quantum-like, $|\vec{E}|^2$ excitation term reproduces the predictions of the photoelectric effect. The slight asymmetry originates from the density of states $\rho_e(\mathcal{E})$ [29]. Some earlier papers, e.g., [30] (and potentially, also [23][31])

used excitation rates similar to those of Eq. (S9) to *qualitatively* describe the steady-state "hot" electron density. However, such a qualitative estimate is appropriate *only* in case all other terms in the underlying equation are energy-independent. Clearly, from Fig. S1, this is not generically the case. As explained in more detail in [32], this approach does not describe correctly the electron distribution near the Fermi energy, but it can describe the electron distribution correctly far from the Fermi energy (via multiplication by the $e-e$ collision time). Unfortunately, the former effect is orders of magnitude more important.

Note that in our approach, we effectively assume that momentum is conserved for all transitions. A more accurate description requires one to distinguish between the electron states according to their momentum, as done e.g., in [6] for a continuum of electron states and in [9, 10] for discretized electron states. However, it is worth noting in this context that the numerical results in [10] show that when considering an ensemble of many nanoparticles with a variation in shape (up to 40%), quantization effects nearly disappear even for a 2nm (spherical) particle. Indeed, the analytical result (red lines in Figs. 4 and 5 of [10]) for the high-energy carrier generation rate, obtained by taking the continuum state limit, is very similar to the exact discrete calculation averaged over the particle sizes. This shows that neglecting the possibility of momentum mismatch (which is the effective meaning of avoiding the energy state quantization, as essentially done in our calculations) provides a rather tight upper limit estimate. Having said that, we bear in mind that quantization effects may still be relevant in highly regular nanoparticle distributions, ordered nanoparticle arrays or single nanoparticle experiments.

B. The $e-ph$ collision term

In [5], the rate of change of f due to $e-ph$ collisions is derived from the Bloch-Boltzmann-Peierls form [1, 33]'[34], giving

$$\left(\frac{\partial f}{\partial t}\right)_{e-ph} = -\frac{\mathcal{X}^2\sqrt{m^*_{eff}}}{4\pi\rho\sqrt{2\mathcal{E}}}\frac{1}{\hbar v_{ph}}\int_0^{\mathcal{E}_D} d\mathcal{E}_{ph}\mathcal{E}_{ph}^2\{f(\mathcal{E})\left([1-f(\mathcal{E}+\mathcal{E}_{ph})]n(\mathcal{E}_{ph})+[1-f(\mathcal{E}-\mathcal{E}_{ph})][n(\mathcal{E}_{ph})+1]\right)$$
$$-[1-f(\mathcal{E})]\left[f(\mathcal{E}+\mathcal{E}_{ph})[n(\mathcal{E}_{ph})+1]+f(\mathcal{E}-\mathcal{E}_{ph})n(\mathcal{E}_{ph})\right]\}. \tag{S10}$$

Here, $\mathcal{X}\sim 2\mathcal{E}_F/3$ is the effective deformation potential [5], ρ is the material density and m^*_{eff} is the effective electron mass [35]. For simplicity, we further assume that the phonon system is in equilibrium, so that $n(\mathcal{E}_{ph}) = n^T(\mathcal{E}_{ph};T_{ph}) = \left(e^{\frac{\mathcal{E}_{ph}}{k_BT_{ph}}}-1\right)^{-1}$ is the Bose-Einstein

distribution function where $\mathcal{E}_{ph}$ is the phonon energy and T_{ph} is the phonon temperature. Eq. (S10) relies on the Debye model [36], namely, a linear dispersion relation for the phonons is assumed, $\mathcal{E}_{ph} = v_{ph}\hbar|q|$, where v_{ph} is the speed of sound ($\cong$ 3650m/s in Ag) and q is the phonon momentum. Beyond the Debye energy, $\mathcal{E}_D = k_B T_D \cong 0.015$eV for Ag, the density of phonon states vanishes. Previous work emphasized the *in*sensitivity of the non-equilibrium dynamics to the phonon density of states and dispersion relations, thus, justifying the adoption of this simple model [5, 37] and the neglect of the phonon non-equilibrium. More advanced models that account also for the possible non-equilibrium of the lattice exist (see e.g., in [7, 38]) but are relatively rare.

The two terms associated with $f(\mathcal{E} + \mathcal{E}_{ph})$ describe phonon absorption, whereas the two terms associated with $f(\mathcal{E} - \mathcal{E}_{ph})$ describe phonon emission. Fig. S1(b) shows the energy dependence of these four different processes described by Eq. (S10) for $T_e - T_{ph} = 0.2$°K, neglecting the small non-thermal part of the distribution (justified a-posteriory). For this temperature difference, an estimate based on the relaxation time approximation for $e - ph$ collisions allows us to relate the magnitude of each term ($\sim 10^{12}/sec$) to a collision rate of $\sim$ 10fs, in accord with the value sometimes adopted within this context [25]. However, since these four processes compete with each other, the resulting total change of the distribution due to $e-ph$ collisions is several orders of magnitude slower. Overall, one can see that (S10) has a rather symmetric, $\sim \hbar\omega_D$-wide Lorentz-like lineshape. For $T_e > T_{ph}$, the rate is negative (positive) above (below) the Fermi energy, reflecting the higher likelihood of phonon emission processes, i.e., that energy is transferred from the electrons to the phonons. In order to see this more clearly, we can calculate the rate of energy transfer between the electrons and phonons by multiplying by $\mathcal{E}\rho_e(\mathcal{E})$ and integrating over all electron energies. The resulting integral, defined as $W_{e-ph} \equiv -\int_0^\infty \mathcal{E}\rho_e(\mathcal{E}) \left(\frac{\partial f}{\partial t}\right)_{e-ph} d\mathcal{E}$, is hardly distinguishable from its thermal counterpart, $W^T_{e-ph} \equiv -\int_0^\infty \mathcal{E}\rho_e(\mathcal{E}) \left(\frac{\partial f^T}{\partial t}\right)_{e-ph} d\mathcal{E}$, which is usually represented by $G_{e-ph}(T_e - T_{ph})$ [33]. For $T_e > T_{ph}$, the factor $\mathcal{E}\rho_e(\mathcal{E})$ weighs favourably the region above the Fermi energy, such that W_{e-ph} and W^T_{e-ph} are positive. In [37], an ab-initio, parameter-free derivation of the electron-phonon coupling coefficient based on density functional theory found $G_{e-ph} \sim 3 \cdot 10^{16} W/m^3K$ for Ag, in agreement with values found in previous works [5, 7, 33, 39–41], and with a negligible temperature-dependence, up to about 3000°K.

We note that our approach accounts for the mutual effect $e - e$ collisions have on $e - ph$ collisions [4], since $e - ph$ collisions are treated by the f-dependent rate (S10) (rather than

within the relaxation time approximation).

C. The $e-e$ collision term

1. The $e-e$ collision rate

The rate of $e-e$ collisions near thermal equilibrium is usually slower than the $e-ph$ collision rate (order of picoseconds) since they involve only deviations from the independent electron approximation [2]. However, away from thermal equilibrium, the $e-e$ collision rates of high-energy non-thermal electrons increase substantially and can become comparable to the $e-ph$ collision rate or even faster (see Fig. S1(c)). Specifically, by Landau's Fermi liquid Theory (FLT) [42], the (effective) $e-e$ collision rate is given by

$$\tau_{e-e}^{-1}(\mathcal{E}) = K\left[(\pi k_B T_e)^2 + (\mathcal{E} - \mathcal{E}_F)^2\right], \tag{S11}$$

where $K = {m_{eff}^*}^3/8\pi^4\hbar^6 W_{e-e}$ is the characteristic $e-e$ scattering constant that contains the angular-averaged scattering probability W_{e-e} and the effective mass of the electron, m_{eff}^*; for Au and Ag, $K = 2\cdot 10^{14}/eV^2\ s$ [4]. Similar variations of this expressions within a continuum of states description were used e.g., in [5–7] in the context of ultrafast illumination. The more recent calculations of the $e-e$ collision rate within a discretized electron energy description, e.g., in [9, 16] retrieved this functional dependence. Experimental data obtained via two photon photo-emission measurements are found in excellent agreement with the Fermi liquid based expression (S11), see discussion in [7][43].

2. Energy conserving relaxation time approximation

Since $e-e$ collisions are elastic (and within the approximation adopted here, also isotropic) [3], we can adopt the relaxation time approximation for sufficiently small deviation from equilibrium, and write

$$(\Delta_\tau f)_{e-e} = -\frac{f(\mathcal{E}, T_e, T_{ph}) - f^T(\mathcal{E}, T_e)}{\tau_{e-e}(\mathcal{E})}. \tag{S12}$$

However, we note that the regular $e-e$ term does not conserve the energy of the electron system as a whole (although it is supposed to, by the elastic nature of $e-e$ collisions). As a remedy, we introduce a term $\mathcal{F}_{e-e}(\mathcal{E})$, defined by the condition

$\int_0^\infty \mathcal{E}\rho_e(\mathcal{E})\left[(\Delta_\tau f)_{e-e} + \mathcal{F}_{e-e}(\mathcal{E})\right] d\mathcal{E} = 0$. This additional term ensures that the electron energy, defined as $\mathcal{U}_e \equiv \int_0^\infty \mathcal{E}\rho_e(\mathcal{E}) f(\mathcal{E}, T_e, T_{ph}) d\mathcal{E}$, is conserved. Such a term is regularly included in Boltzmann models of fluid dynamics, where it is known as the Lorentz term [44], but to our knowledge, was not employed in the context of illuminated metal nanostructures [45]. Thus, overall, we have

$$\left(\frac{\partial f}{\partial t}\right)_{e-e} = (\Delta_\tau f)_{e-e} + \mathcal{F}_{e-e}(\mathcal{E}). \tag{S13}$$

The absence of this term in previous steady-state derivations of the electron distribution (e.g., in [10, 13, 14]) mean that energy is not conserved in these studies; Nevertheless, this specific effect is relatively small.

3. Comparison between different scattering time functions

The results in the main text were obtained using an $e-e$ collision time of the form (S11). The coefficient K was found using a fit to the calculations of Ref. [9]. In addition, we performed the same calculation with a phenomenological scattering time of the form $\tau_{e-e}(\mathcal{E}) = e^{(1/(a+b\mathcal{E})}$, with a = 0.08585, b = 0.1278 eV^{-1} (which decays slightly faster than the usual energy-dependence of the FLT); this seems to fit the data of Ref. [9] better. In Fig. S2 we show the original data of Ref. [9, Fig. 6] and the fits to the standard FLT form (S11) and the phenomenological form.

In Fig. S3 we show the "hot" electron distribution evaluated with these two forms for the $e-e$ collision time, the FLT one and the phenomenological one. As can be seen, while the distributions are slightly different, the difference is essentially quantitative. The electron and phonon temperatures were found to be identical for the 2 expressions for τ_{e-e} (within our numerical accuracy for the 2 cases).

D. Evaluating the power density for different process

The formalism presented in this manuscript allows us to evaluate the power density that goes into heating the electrons and phonons, and the power that goes into generating non-thermal carriers.

To evaluate this, we start with the general expression for the total energy of the electron system, $\mathcal{U}_e$ defined above. Formally taking the time derivative gives the total power output

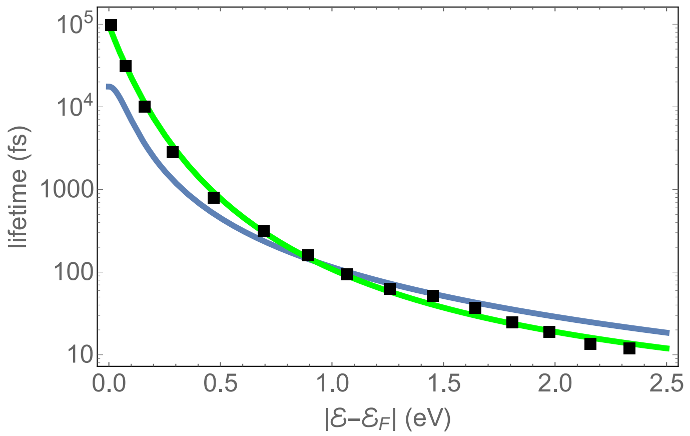

FIG. S2: (Color online) The $e-e$ scattering time, evaluated by averaging over the data of from Ref. [9] (black squares). Solid blue line is a fit to the standard (Fermi liquid) collision time (S11), and the solid green line are fits to the phenomenological form defined in the text.

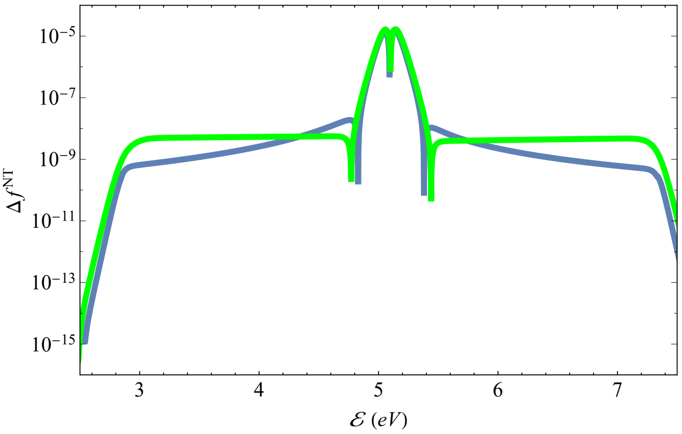

FIG. S3: (Color online) Non-equilibrium electron distributions (see main text) for the two forms of $e-e$ scattering time, showing only a qualitative difference.

of the electron system (which, at steady-state, vanishes by definition),

$$\frac{d\mathcal{U}_e}{dt} = \int \mathcal{E}\rho_e(\mathcal{E})\left(\frac{\partial f}{\partial t}\right) d\mathcal{E}. \tag{S14}$$

From Eq. (1), one can formally break $\left(\frac{\partial f}{\partial t}\right)$ into different contributions. Plugging these contributions into Eq. (S14) the power that goes into the difference energy channels. Specifically, substituting $\left(\frac{\partial f}{\partial t}\right)_{ex}$ gives the expression for the total power that is pumped into the electronic system by the photons.

Similarly, our formalism provides a natural way to distinguish between thermal and non-thermal contributions, since the steady-state distribution is naturally *a-priori* defined as

$f(\mathcal{E}) = f^T(\mathcal{E}, T_e) + f^{NT}(\mathcal{E})$. The first term is a thermal distribution with the (elevated) steady-state electron temperature, and the second term is the non-thermal distribution. Thus, substituting these into the expression for power gives the power W^T that goes into the thermal part of the electron distribution (i.e., that goes into electron heating) and the power W^{NT} that goes into generating non-thermal carriers, namely,

$$W^T = \int \mathcal{E}\rho_e(\mathcal{E}) \left[\left(\frac{\partial f}{\partial t} \right)_{ex} \right]_{f=f^T} d\mathcal{E}, \tag{S15}$$

$$W^{NT} = \int \mathcal{E}\rho_e(\mathcal{E}) \left[\left(\frac{\partial f}{\partial t} \right)_{ex} \right]_{f=f^{NT}} d\mathcal{E}. \tag{S16}$$

II. ELECTRON TUNNELING FROM THE NANOPARTICLE

The use of plasmonic naonparticles for applications requires that the "hot" electrons tunnel out of the nanoparticle in order to perform some function, be it tunneling into a molecular orbital for photocatalysis or across a Schottky barrier with a semiconductor for detection. The underlying assumption of much of the literature is that if such a process occurs at a given energy, then, the efficiency of the process will be proportional to the electron distribution at that energy. For example, in discussing tunneling across a barrier, then the eficiency of the process will be simply an integral over the electron distribution function over energies higher than the barrier energy (with some weight). Similarly, for tunneling into a molecular level, the efficiency will be proportional to the electron distribution at that energy.

In a recent paper [32] we have shown that this is indeed the case for photo-catalysis, as long as the tunneling time is long compared to all other timescales, most importantly the $e-e$ scattering time. The argument relies on evaluating the distribution function in the presence of a tunneling term. We start by describing a tunneling term of the form $g(\varepsilon)f(\varepsilon)$, where f is the distribution, and $g(\varepsilon)$ is some kernel, describing the tunneling rate per energy. Importantly, (i) g is independent of the distribution and (ii) is localized at energies far from the Fermi energy, where the distribution is small (in fact, it could be a step-like function, for example if there is tunneling through a Schottky barrier, but here we have in mind photo-catalysis. The explanations below actually apply for both classes of applications).

Now, assuming that we know the steady-state distribution $f_0(\varepsilon)$, we look for a correction to it, $f = f_0 + f_1$. The next step is to linearize the bare Liouvillian (i.e. the right-hand-side

of the Boltzmann equation without the tunnelling term). Then, for the steady-state we have

$$0 = \alpha(\varepsilon) f_1(\varepsilon) + g(\varepsilon)(f_0(\varepsilon) + f_1(\varepsilon)), \quad \text{(S17)}$$

where $\alpha(\varepsilon)$ is the linearization term. This equation can easily be solved to give $f_1 = \frac{g}{g+\alpha} f_0$. Now, as long as the dependence of α on energy is rather weak (which is indeed the case for both $e-e$ collision time and the excitation term), and the dependence of f_0 itself on ε is also weak, the correction to the distribution function is simply proportional to the tunneling term $g(\varepsilon)$.

In order to test this (rather simple) estimate, we ran our calculation with an additional tunneling term of the form $-\gamma_T g(\varepsilon) f(\varepsilon)$, where $\gamma_T = 10^{13}$Hz and 10^{15}Hz, corresponding to a slow (100 femtosecond) and fast (few femtosecond) tunneling time (which is extremely fast, as realistic tunneling times were shown to be as short as 100 fs only in the best case scenario, see e.g., [46]); g is centered at ≈ 1.5eV above the Fermi energy and has an energy width of a few hundreds of meV. In Fig. S4 we show the electron distribution with the tunneling terms, and the approximation (S17). As can be seen, for $\gamma_T = 10^{13}$Hz the approximation above is excellent.

Even more surprising and interesting, while for $\gamma_T = 10^{15}$Hz there should be a difference (because formally we are outside the regime of the approximation), still the approximation seems very good. The conclusion we draw from this calculation is that, in principle, and over a wide range of parameters (and physical processes), knowledge of the bare distribution function (i.e., evaluated without a tunneling terms) provides an excellent indication to the performance of the "hot"-electron system as a functional device.

III. PRACTICAL CONSIDERATIONS

In order to avoid limiting the generality of our results, we did not indicate throughout the manuscript details of a specific nanostructure. In this SI Section, we discuss what needs to be done in order to apply our theory to a specific experimental configuration. For simplicity, we discuss nanospheres; extension of the discussion to other particle shapes is possible.

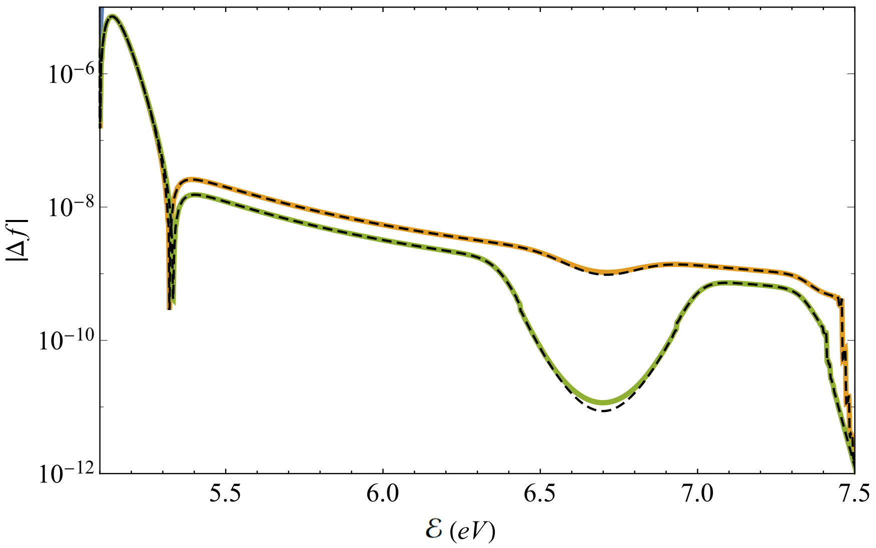

FIG. S4: Non-equilibrium electron distribution with a tunneling term described in the text above, for $\gamma_T = 10^{13}$Hz (orange line) and 10^{15}Hz (green line). The dashed lines are the approximations (S17).

A. Local field

Throughout the manuscript, we treated $|\vec{E}|$ as a parameter representing the *local* field [47]. In order to evaluate the non-thermal carrier density for an actual nanostructure configuration and illumination pattern, one needs to solve the Maxwell equations for the given configuration (for example, $\vec{E} = [3\epsilon_h/(2\epsilon_h + \epsilon_m)]\,\vec{E}_{inc}$ for a small sphere illuminated uniformly) and apply our formulation *locally*, i.e., for each point in the nanostructure independently; this procedure was adopted in [10] and was complemented by surface/volume averaging. In that respect, the role of surface plasmon resonances in promoting "hot" carrier generation is obvious - at resonance, the local electric fields are enhanced, hence, the electron system is driven more strongly away from equilibrium.

For weak electric fields, like used in the current work and essentially in all relevant experiments (see e.g., [48, 49], the distribution and temperatures can then be readily determined. For small spherical metal nanoparticles, the temperature(s) are uniform [20, 21]. The majority of previous theoretical studies relied on these same assumptions (e.g., [9, 13, 23]).

For more complicated geometries, or for bigger nanostructures, the field may not be uniform. Nevertheless, the gradients of the electric fields are usually assumed to have a small effect on the electron distribution. The non-uniformity of the temperature is negligible, due

to the relatively high thermal conductivity of the metal [20, 21]. Due to these reasons, these gradients were neglected in all previous studies; we adopt the same approach here. For higher fields, the optical and thermal properties of the metal may change due to the rise in temperature, requiring a fully self-consistent solution of the coupled Maxwell, Boltzmann and heat equations. Such a treatment is left to a future study.

B. Particle size

The size of the particle affects the field relatively weakly for sufficiently small size (for which the quasi-static approximation holds). However, as well-known [20], the nanoparticle temperature depends strongly on the particle size; for example, for nano-spheres, it grows quadratically with the radius a. In our formulation, this effect is accounted for via the value of the phonon-environment coupling, G_{ph-env}, which is usually calculated from first principles via molecular dynamics simulations, see e.g., [50–53]. Overall, it scales inversely with the surface area [6] for nano-spheres; this is equivalent to assuming the total heat conductance to the environment is proportional to the particle surface area; this scaling facilitates estimates for non-spherical particles.

As pointed out in the main text, we have carried out additional calculations to demonstrate the dependence of electron distribution and temperatures on the particle size via G_{ph-env}. The original results (appearing in the main text figures; 5nm particle size; $G_{ph-env} = 5 \times 10^{14}$ W/m^{3}K [54]) can now be compared to results for a particle which is 10 times bigger (50nm; $G_{ph-env} = 5 \times 10^{12}$ W/m^{3}K). In Fig. S5(a), we plot the electron and phonon temperatures as a function of intensity for these two cases. As can be observed, the electron temperature rise is $\sim$ 100-fold larger for the larger particle (compare to Fig. 3), namely, about 30K. However, the difference between the electron and phonon temperatures is roughly the same; indeed, it can be shown analytically to be proportional to the incoming intensity which is the same for both sets of simulations.

In Fig. S5(b) we plot the electron non-equilibrium distribution (specifically, the absolute value of the deviation of the electron distribution from the Fermi distribution, $|\Delta f|$) for the two particle sizes and for two illumination levels, $|\vec{E}|^2 = 1.4 \times 10^6$, $1.4 \times 10^9 (\mathrm{V/m})^2$. It is readily seen that the only deviations between the large and small particle cases are at the vicinity of the Fermi energy, but the non-thermal parts of the distributions (i.e., further away

from $\mathcal{E}_F$, where $\Delta f^{NT} \sim \Delta f$) are *insensitive* to the particle size. In particular, we find that the efficiency of non-thermal high-energy electron generation is independent of particle size, but the overall heating scales as a^2, in agreement with the single-temperature (classical) heat equation. Such correspondence is absent in the simulations in [10] [55]. This also means that smaller particles give rise to a higher relative efficiency of non-thermal carrier generation. This prediction should motivate a careful, single particle study that will enable one to verify this prediction vs. potentially contradicting claims based on measurements from macroscopic nanoparticle suspensions.

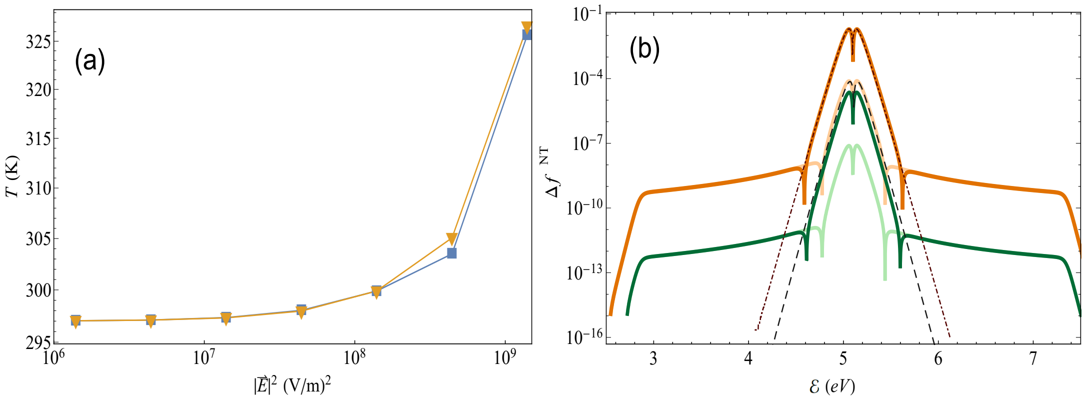

FIG. S5: (a) Electron (yellow) and phonon (blue) temperatures as a function of $|\vec{E}|^2$, for a system with a host thermal conductivity $G_{ph-env} = 5 \times 10^{12}$ W/m^{3}K, two orders of magnitude smaller than that employed in the simulations shown in Figs. 1-4 of the main text. Correspondingly, the temperature rise is much larger, as well as the difference between the electron and phonon temperatures. (b) Deviation of the non-equilibrium distribution from the thermal distribution for low host thermal conductivity and two intensities, $|\vec{E}|^2 = 1.4 \times 10^6, 1.4 \times 10^8 (\text{V/m})^2$ (dark green and dark orange lines, respectively). For comparison, the distributions from the high G_{ph-env} values used in Fig. 1 are also plotted (light green and light orange solid lines). The dashed black lines show the differences between simple Fermi functions with $T = 297.9$K (which is the electron temperature corresponding to the dark green line) and $T = 325$K (which is the electron temperature corresponding to the dark orange line) to a Fermi function at ambient temperature of 297K.

The results of Fig. S5(b) can be also interpreted in terms of the dependence of the non-thermal distribution on the host thermal conductivity. Indeed, the rate of energy density transfer to the environment G_{ph-env} is also proportional to the thermal conductivity of the

host [6]. Thus, the different curves in Fig. S5(a) can be also associated with a system with a host thermal conductivity which is two orders of magnitude lower than the one presented in the main text. As for the larger nanoparticle, the electron temperature rise and the difference between the electron and phonon temperatures, are higher, as expected - indeed, the heat flows away from the nanoparticle much more slowly for the larger nanoparticle. This shows, as stated in the main text, that if "hot" electrons play a dominant role in some experiment (e.g. in photo-catalysis), then, the experimental results should be unaffected by a change of host. Conversely, if the results *are* affected by a change of host material (as observed e.g., in [48, 49]), then it is not likely that the reason for that is the number of "hot" electrons, but rather due to a thermal effect, or an altogether different chemical effect; for a detailed discussion, see also [56].

C. Surface scattering and quantum size effects

If one is interested in even smaller nanoparticles, then, within the energy state continuum description used in the current work, it may be necessary to account also for $e - surface$ collisions (the so-called "quantum size effects"), as noted as early as in [57]. As this effect does not involve conservation of electron momentum, it can be accounted for in our formulation by adding a relaxation time like term, $(f - f^T)/\tau$, where τ is the time scale for these collisions which can be as fast as a few hundreds of femtoseconds in the case of a metal surface with atomic roughness [46]; accordingly, it is practically negligible with respect to the $e - e$ and $e - ph$ collision rates. Depending on the nature of the $e - surface$ collisions, one may want to include/exclude them from the conservative term, $\mathcal{F}_{e-e}(\mathcal{E})$.

However, it should be noted that in more advanced models where the energy states are discretized (such that $e - surface$ collisions are accounted for inherently), e.g., [9, 10], the electronic states and the phononic states are extended throughout the bulk, and no "surface states" appear. One thus expects that in such calculations there will be no separate contribution from $e - surface$ collisions. In fact, in [9, 16] it was shown that the electron collision time is independent of the nanoparticle size. All these results indicate that unlike previous claims [58] "quantum size effects" have at most a small quantitative effect on the non-thermal carrier generation efficiency. This result was corroborated in [10], see discussion

at the end of Section I A.

[1] J. M. Ziman. *Principles of the theory of solids.* Cambridge University Press, 1972.

[2] N. W. Ashcroft and N. D. Mermin. *Solid state physics.* Brooks/Cole, 1976.

[3] M. Lundstrom. *Fundamentals of carrier transport.* Addison-Wesley, 1990.

[4] R. H. M. Groeneveld, R. Sprik, and A. Lagendijk. Femtosecond spectroscopy of electron-electron and electron-phonon energy relaxation in Ag and Au. *Phys. Rev. B*, 51:11433–11445, 1995.

[5] N. Del Fatti, C. Voisin, M. Achermann, S. Tzortzakis, D. Christofilos, and F. Valleé. Nonequilibrium electron dynamics in noble metals. *Phys. Rev. B*, 61:16956–16966, 2000.

[6] P. Grua, J. P. Morreeuw, H. Bercegol, G. Jonusauskas, and F. Valleé. Electron kinetics and emission for metal nanoparticles exposed to intense laser pulses. *Phys. Rev. B*, 68:035424, 2003.

[7] L. D. Pietanza, G. Colonna, S. Longo, and M. Capitelli. Non-equilibrium electron and phonon dynamics in metals under femtosecond laser pulses. *Eur. Phys. J. D*, 45:369–389, 2007.

[8] M. Kornbluth, A. Nitzan, and T. Seidman. Light-induced electronic non-equilibrium in plasmonic particles. *J. Chem. Phys.*, 138:174707, 2013.

[9] J. R. M. Saavedra, A. Asenjo-Garcia, and F. Javier Garcia de Abajo. Hot-electron dynamics and thermalization in small metallic nanoparticles. *ACS Photonics*, 3:1637–1646, 2016.

[10] L. V. Besteiro, X.-T. Kong, Z. Wang, G. Hartland, and A. O. Govorov. Understanding hot-electron generation and plasmon relaxation in metal nanocrystals: Quantum and classical mechanisms. *ACS Photonics*, 4:2759–2781, 2017.

[11] D. Vasileska and S. M. Goodnick (eds.). Nano-electronic devices: Semiclassical and quantum transport modeling. *Physics Reports*, 478:71–120, 2009.

[12] A. K. Chattah and M. O. Cáceres. Computing the quantum Boltzmann equation from a Kossakowski-Lindblad generator. In: O. Descalzi, J. Martínez, E. Tirapegui (eds.), Instabilities and Nonequilibrium Structures VII & VIII. Nonlinear Phenomena and Complex Systems. 8, 2004.

[13] A. O. Govorov, H. Zhang, and Y. K. Gun'ko. Theory of photoinjection of hot plasmonic carriers from metal nanostructures into semiconductors and surface molecules. *J. Phys. Chem.*

C, 117:16616–16631, 2013.

[14] H. Zhang and A. O. Govorov. Optical generation of hot plasmonic carriers in metal nanocrystals: The effects of shape and field enhancement. *J. Phys. Chem. C*, 118:7606–7614, 2014.

[15] Note that we ignore here the difference between the Fermi energy and the chemical potential; we verified in simulations that the difference between them is truly negligible in all cases we studied.

[16] A. M. Brown, R. Sundararaman, P. Narang, W. A. Goddard, and H. A. Atwater. Nonradiative plasmon decay and hot carrier dynamics: Effects of phonons, surfaces, and geometry. *ACS Nano*, 10:957–966, 2016.

[17] J. Khurgin, W.-Y. Tsai, D. P. Tsai, and G. Sun. Landau damping and limit to field confinement and enhancement in plasmonic dimers. *ACS Photonics*, 4:2871–2880, 2017.

[18] M. Bernardi, J. Mustafa, J. B. Neaton, and S. G. Louie. Theory and computation of hot carriers generated by surface plasmon polaritons in noble metals. *Nature Commun.*, 6:7044, 2015.

[19] Notably, this is in contrast to the claims in [58] which were not supported by evidence.

[20] G. Baffou, R. Quidant, and F. J. Garcia de Abajo. Nanoscale control of optical heating in complex plasmonic systems. *ACS Nano*, 4:709–716, 2010.

[21] I. W. Un and Y. Sivan. Size-dependence of the photothermal response of a single metal nanosphere. *https://arxiv.org/abs/1907.01255*, 2019.

[22] A. Govorov and H. Zhang. Kinetic density functional theory for plasmonic nanostructures: Breaking of the plasmon peak in the quantum regime and generation of hot electrons. *J. Phys. Chem. C*, 119:6181–6194, 2015.

[23] A. Manjavacas, J. G. Liu, V. Kulkarni, and P. Nordlander. Plasmon-induced hot carriers in metallic nanoparticles. *ACS Nano*, 8:7630–7638, 2014.

[24] S. T. Sundari, S. Chandra, and A. K. Tyagi. Temperature dependent optical properties of silver from spectroscopic ellipsometry and density functional theory calculations. *J. Appl. Phys.*, 033515:114, 2013.

[25] M. Dressel and G. Grüner. *Electrodynamics of solids - optical properties of electrons in matter*. Cambridge University Press, 2002.

[26] A. Marini, A. Ciattoni, and C. Conti. Out-of-equilibrium electron dynamics of silver driven by ultrafast electromagnetic fields a novel hydrodynamical approach. *Faraday Discussions*,

214:235, 2019.

[27] B. Rethfeld, A. Kaiser, M. Vicanek, and G. Simon. Ultrafast dynamics of nonequilibrium electrons in metals under femtosecond laser irradiation. *Phys. Rev. B*, 65:214303, 2002.

[28] J. D. Jackson. *Classical electrodynamics.* Wiley & Sons, 3rd edition, 1998.

[29] This asymmetry may grow if the energy dependence of D_J will be taken into account.

[30] T. Gong and J. N. Munday. Materials for hot carrier plasmonics. *Optical Materials Express*, 5:2501, 2015.

[31] In that paper, a similar calculation was done, namely, of the "hot" electron excitation rate (rather than their density); however, the results were not shown on a logarithmic scale, hence, it is difficult to observe the similarity.

[32] Y. Sivan, I. W. Un, and Y. Dubi. Assistance of plasmonic nanostructures to photocatalysis - just a regular heat source. *Faraday Discussions*, 214:215–233, 2019.

[33] P. B. Allen. Theory of thermal relaxation of electrons in metals. *Phys. Rev. Lett.*, 59:1460–1463, 1987.

[34] This expression does not include Umklapp collisions.

[35] In our simulations, we used the values for these parameters as given in [5]. However, it should noted that the value they quote for ρ might have involved a typo, which in turn, might have been adjusted via the value of $\mathcal{X}$. Either way, the overall value obtained for the cumulative $e-ph$ term (G_{e-ph}) is found to be in excellent agreement with the value computed in several other studies.

[36] This is justified for noble metals, such as Ag, where only acoustic phonons are present. Assuming that these phonon modes are distinct and excluding Umklapp processes, only the longitudinal phonon acoustic mode is coupled to the electron gas.

[37] A. M. Brown, R. Sundararaman, P. Narang, W. A. Goddard, and H. A. Atwater. Ab initio phonon coupling and optical response of hot electrons in plasmonic metals. *Phys. Rev. B*, 94:075120, 2016.

[38] V. V. Baranov and V. V. Kabanov. Theory of the electron relaxation in metals excited by an ultrashort optical pump. *Phys. Rev. B*, 84:125102, 2014.

[39] M.I. Kaganov, I.M. Lifshitz, and L.V. Tanatarov. Relaxation between electrons and crystalline lattices. *Sov. Phys. JETP*, 4:173–178, 1957.

[40] P.E. Hopkins. Contributions of inter and intra-band excitations to electron heat capacity and

electron-phonon coupling in noble metals. *J. Heat Transfer*, 132:014504, 2010.

[41] A. Giri and P. E. Hopkins. Transient thermal and nonthermal electron and phonon relaxation after short-pulsed laser heating of metals. *J. of Appl. Phys.*, 118:215101, 2015.

[42] P. Coleman. *Introduction to many body physics.* Cambridge University Press, 2015.

[43] It should be noted, however, that some earlier studies (e.g., [4]) employed a different expression for τ_{e-e} which incorporates a strong asymmetry with respect to the Fermi energy, based on the famous expression derived in [59, Pines & Nozieres]. However, Coleman [42] showed that the Pines & Nozieres expression is, in fact, unsuitable for our purposes and that the symmetric parabolic dependence of the collision rate on the energy difference with respect to the Fermi energy (as in [9, 10, 16]) is in fact the correct one. Indeed, the Pines & Nozieres traces the collision dynamics of a single electron, rather than the relaxation dynamics of the distribution as a whole; in other words, it accounts for scattering of electrons from a certain electronic state $\mathcal{E}$, but ignores scattering *into* that energy state, a process which cancels out the dependence of the scattering rate on the Fermi function.

[44] E. H. Hauge. Exact and Chapman-Enskog solutions of the Boltzmann equation for the Lorentz model. *The Physics of Fluids*, 13:1201, 1970.

[45] However, we note that in models that rely on the complete $e-e$ scattering integral (e.g., see examples in the context of ultrafast illumination [4–7]), the electron energy is conserved, so that the Lorentz term is not necessary.

[46] M. Grajower, J. Khurgin, and U. Levy. The role of surface roughness in plasmonic-assisted internal photoemission schottky photodetectors. *ACS Photonics*, 5:4030–4036, 2018.

[47] Also note that throughout the manuscript we avoid specifying the local intensity, as it is a somewhat improper quantity to use when discussing metals. Indeed, the negative real part of the permittivity causes the fields within the metal to be primarily evanescent, hence, not to carry energy (such that the Poynting vector, hence, intensity vanish, at least in the absence of absorption). Instead, we use the local density of electromagnetic energy, by specifying the local electric field, which is easy to connect to the incoming field.

[48] S. Mukherjee, F. Libisch, N. Large, O. Neumann, L. V. Brown, J. Cheng, J. Britt Lassiter, E. A. Carter, P. Nordlander, and N. J. Halas. Hot electrons do the impossible: Plasmon-induced dissociation of H_2 on Au. *Nano Lett.*, 13:240–247, 2013.

[49] S. Mukherjee, L. Zhou, A. Goodman, N. Large, C. Ayala-Orozco, Y. Zhang, P. Nordlander,

and N. J. Halas. Hot-electron-induced dissociation of H_2 on gold nanoparticles supported on SiO_2. *J. Am. Chem. Soc.*, 136:64–67, 2014.

[50] J. C. Duda, C.-Y. P. Yang, B. M. Foley, R. Cheaito, D. L. Medlin, R. E. Jones, and P. E. Hopkins. Influence of interfacial properties on thermal transport at gold:silicon contacts. *Appl. Phys. Lett.*, 102:081902, 2013.

[51] H.-K. Lyeo and D. G. Cahill. Thermal conductance of interfaces between highly dissimilar materials. *Phys. Rev. B*, 73:144301, 2006.

[52] S. Merabia, S. Shenogin, L. Joly, P. Keblinski, and J.-L. Barrata. Heat transfer from nanoparticles: A corresponding state analysis. *Proc. Nat. Acad. Sci. U.S.A*, 106:15113–15118, 2009.

[53] X. Chen, A. Munjiza, K. Zhang, and D. Wen. Molecular dynamics simulation of heat transfer from a gold nanoparticle to a water pool. *J. Phys. Chem. C*, 118:1285 – 1293, 2014.

[54] D. T. Owens, C. Fuentes-Hernandez, J. M. Hales, J. W. Perry, and B. Kippelen. A comprehensive analysis of the contributions to the nonlinear optical properties of thin Ag films. *Journal of Applied Physics*, 107:123114, 2010.

[55] In [10], the electron temperature was not evaluated self-consistently, as in our formulation, but rather, it was set by hand and referred to as an "effective" temperature; no discussion of the choice of values was given. Unfortunately, the effective electron temperature values were set to $\approx 1300K$ (0.1 eV for a 4nm NP), whereas the single-temperature (classical) calculation for this configuration shows that the temperature rise should be $\approx 0.13K$. In addition, the scaling of the effective temperature used in [10] violates the classical a^2 scaling; in fact, it showed an inverse proportionality to the NP size (specifically, the effective temperature of a 24nm NP was ≈ 520K (0.04eV)). Claims in [Govorov & Besteiro, ArXiv 2019] on the emergence of quantum effects in this context are questionable, due the relatively large size of the NPs studied in this case, see also the discussion at the end of Section I A.

[56] Y. Sivan, I. W. Un, and Y. Dubi. Thermal effects - an alternative mechanism for plasmonic-assisted photo-catalysis. *submitted; https://arxiv.org/abs/1902.03169*, 2019.

[57] U. Kreibig and M. Vollmer. *Optical Properties of Metal Clusters.* Springer: Berlin, 1995.

[58] M. L. Brongersma, N. J. Halas, and P. Nordlander. Plasmon-induced hot carrier science and technology. *Nature Nanotech.*, 10:25, 2015.

[59] D. Pines and P. Nozieres. *The theory of quantum liquids.* Benjamin, New York, New York, 1966.
